# Noninvasive mechanical ventilation may be useful in treating patients who fail weaning from invasive mechanical ventilation: a randomized clinical trial

**DOI:** 10.1186/cc6870

**Published:** 2008-04-17

**Authors:** Cristiane E Trevisan, Silvia R Vieira, the Research Group in Mechanical Ventilation Weaning

**Affiliations:** 1 Intensive Care Unit, Hospital de Clínicas de Porto Alegre, Universidade Federal do Rio Grande do Sul, Porto Rua Ramiro Barcelos, 2350, CEP 90035-903, Porto Alegre, RS, Brazil; 2 Universidade Luterana do Brasil, Av. Farroupilha, 8001, CEP 92425-900, Bairro São José, Canoas, RS, Brazil

## Abstract

**Introduction:**

The use of noninvasive positive-pressure mechanical ventilation (NPPV) has been investigated in several acute respiratory failure situations. Questions remain about its benefits when used in weaning patients from invasive mechanical ventilation (IMV). The objective of this study was to evaluate the use of bi-level NPPV for patients who fail weaning from IMV.

**Methods:**

This experimental randomized clinical trial followed up patients undergoing IMV weaning, under ventilation for more than 48 hours, and who failed a spontaneous breathing T-piece trial. Patients with contraindications to NPPV were excluded. Before T-piece placement, arterial gases, maximal inspiratory pressure, and other parameters of IMV support were measured. During the trial, respiratory rate, tidal volume, minute volume, rapid shallow breathing index, heart rate, arterial blood pressure, and peripheral oxygen saturation were measured at 1 and 30 minutes. After failing a T-piece trial, patients were randomly divided in two groups: (a) those who were extubated and placed on NPPV and (b) those who were returned to IMV. Group results were compared using the Student *t *test and the chi-square test.

**Results:**

Of 65 patients who failed T-piece trials, 28 were placed on NPPV and 37 were placed on IMV. The ages of patients in the NPPV and IMV groups were 67.6 ± 15.5 and 59.7 ± 17.6 years, respectively. Heart disease, post-surgery respiratory failure, and chronic pulmonary disease aggravation were the most frequent causes of IMV use. In both groups, ventilation time before T-piece trial was 7.3 ± 4.1 days. Heart and respiratory parameters were similar for the two groups at 1 and 30 minutes of T-piece trial. The percentage of complications in the NPPV group was lower (28.6% versus 75.7%), with lower incidences of pneumonia and tracheotomy. Length of stay in the intensive care unit and mortality were not statistically different when comparing the groups.

**Conclusion:**

The results suggest that NPPV is a good alternative for ventilation of patients who fail initial weaning attempts. NPPV reduces the incidence of pneumonia associated with mechanical ventilation and the need for tracheotomy.

**Trial registration:**

CEP HCPA (02–114).

## Introduction

Several complications may occur during invasive mechanical ventilation (IMV), the most important of which is pneumonia associated with mechanical ventilation [1]. To avoid tracheal intubation and its complications, noninvasive positive-pressure mechanical ventilation (NPPV) has been suggested as an alternative for the management of patients with acute respiratory failure (ARF), particularly during the course of acute pulmonary edema and chronic obstructive pulmonary disease (COPD) [2-8].

One third of IMV time is spent in weaning, defined as the process of gradual removal of mechanical ventilation support toward spontaneous ventilation [9]. Most patients are weaned with no difficulties. However, a significant percentage (5% to 30%) of patients in intensive care units (ICUs) fail spontaneous ventilation trials, characterizing difficult weaning [10]. In the last few years, NPPV has been tested in these situations. Nava and colleagues [11], in a randomized clinical trial, used NPPV or IMV in 50 patients with COPD aggravation who failed spontaneous ventilation trials. The authors found shorter ventilation time and lower mortality with the use of NPPV. Girault and colleagues [12] compared NPPV with pressure support ventilation in 33 COPD patients who failed a 2-hour T-piece trial and found a reduction in total mechanical ventilation time in the NPPV group. However, remaining time in the ICU and survival rates at 3 months were similar in the two groups. Vitacca and colleagues [13] assessed diaphragm energy expenditure (diaphragmatic pressure-time product [PTPdi]), lung resistance and elastance, arterial blood gases, and dyspnea during invasive and noninvasive pressure support ventilation. They found that, in patients with COPD who were not ready to sustain spontaneous breathing, the use of invasive or noninvasive ventilation was equally effective in reducing PTPdi and improving arterial blood gases but that noninvasive ventilation seemed to be better tolerated. In a later study, Ferrer and colleagues [14] suggested that NPPV be assessed as a means to facilitate IMV weaning for patients who failed spontaneous ventilation trials, regardless of the underlying disease. They confirmed the results of the previous study and additionally reported a reduction in remaining hospitalization time and in the need for tracheotomy. Later, a meta-analysis revealed that NPPV facilitates weaning and reduces mortality comparatively to IMV [15]. Quite recently, another two studies showed that the early use of NPPV was efficient in preventing respiratory failure after tracheal extubation in patients at risk for complications and that it reduced mortality in the ICU [16,17]. In all of those trials, most patients had COPD.

Studies assessing NPPV in weaning are still insufficient and generally include a small number of patients. Therefore, questions remain about NPPV benefits in weaning, particularly in heterogeneous groups of patients, which is a usual characteristic of patients admitted to the ICU. Therefore, new controlled and randomized studies are warranted. This study assessed the use of NPPV during weaning from mechanical ventilation in an ICU and compared this procedure with IMV by analyzing cardiac and respiratory parameters, clinical course, and complications.

## Materials and methods

### Population and sample

A randomized clinical trial was conducted from June 2003 to February 2005 with patients in the ICU of Hospital de Clínicas de Porto Alegre (Porto Alegre, Brazil). Patients of any age and both genders were on IMV for more than 48 hours, and their weaning procedures were followed up. Patients who failed at 30 minutes of spontaneous breathing T-piece trial (SBT) were included in the study.

The weaning procedures followed criteria established in the ICU routine: improvement of the cause of ARF that led to the use of ventilation support, correction of arterial hypoxemia (arterial partial pressure of oxygen [PaO_2_] of greater than 60 mm Hg), fraction of inspired oxygen (FiO_2_) of less than or equal to 0.4, and positive end-expiratory pressure (PEEP) of less than or equal to 5 cm H_2_O during pressure support ventilation. All patients were breathing at low levels of pressure support ventilation (less than 12 cm H_2_O). Patients included in the study did not require vasoactive drugs, had an adequate consciousness level (Glasgow coma score of greater than or equal to 13) and cough reflex, and did not require sedation.

Failure or intolerance at 30 minutes of SBT was defined according to one of the following criteria: peripheral oxygen saturation (SpO_2_) measured by pulse oximetry of less than 90% (80% in chronic respiratory failure), respiratory rate (f) of greater than 35 respirations per minute, heart rate (HR) of greater than 140 or less than 50 beats per minute (bpm) (or increase or decrease of greater than 20% in previous mechanical ventilation), and systolic arterial blood pressure of greater than 180 mm Hg or less than 70 mm Hg (or increase or decrease of greater than 20% in previous mechanical ventilation) and rapid shallow breathing index (ratio of f to tidal volume [V_T_], or f/V_T_) of greater than 105. Patients with facial trauma, cranial surgery, recent gastric or esophageal surgery, tracheotomy, excessive respiratory secretion, agitation, or noncooperative behavior were excluded from the study. This study was approved by the Committee on Ethics on Research and Graduate Studies of the Hospital de Clínicas de Porto Alegre.

### Data collection

Patients were included in the study after an informed consent form was signed by a family member or guardian. Patients considered apt to undergo the weaning procedure were submitted to SBT. At that moment, for ICU organizational reasons, patients had already been randomly assigned to one of the ventilatory modes (IMV or NPPV) that would be used in case they failed SBT. Sealed envelopes were used for random assignment.

Before SBT, the following measurements were carried out: arterial blood gases; parameters of IMV such as f; V_T_; minute volume (V_e_); inspiratory pressure peak; PEEP; FiO_2_; PaO_2_/FiO_2 _ratio, and the highest value of three measurements of maximal inspiratory pressure (PI_max_). PI_max_, defined as the maximal inspiratory effort sustained by the patient for 20 seconds, by means of a unidirectional valve, allows for expiration only. Thus, the patient had to make an inspiratory effort in order to trigger the respiratory cycle, and PI_max _was measured at this time [18,19]. This PI_max _was measured using a pressure vacuum meter (Suporte^®^; Porto Alegre, Brazil). At 1 minute and 30 minutes of spontaneous ventilation trial, the following parameters were measured: f, V_T_, V_e_, f/V_T _using a flowmeter (Ohmeda, Madison, WI, USA), HR, systolic (SBP) and diastolic (DBP) blood pressure, and SpO_2 _using a Hewlett-Packard monitor (Hewlett-Packard Company, Palo Alto, CA, USA). If failure occurred before the 30th minute, f, HR, SpO_2_, and SBP and DBP were measured at the time of failure. If the patient failed SBT, he/she was included in the group previously defined by random assignment. Patients in the experimental group were extubated and placed on NPPV, whereas the other patients (the control group) returned to IMV, which was classified as the conventional treatment. The group on NPPV (the experimental group) was extubated after having rested in the mechanical ventilation for 30 minutes in the experimental group. Immediately after tracheal extubation, spontaneous ventilation mode using a bi-level NPPV support unit (Respironics, Synchrony, or S model; Respironics, Inc., now part of Royal Philips Electronics N.V., Amsterdam, The Netherlands) was used. Inspiratory positive airway pressure was delivered according to patient tolerance and varied from 10 to 30 cm H_2_O.

Expiratory positive airway pressure was set at sufficient gas exchange maintenance level and FiO_2 _was set according to an SpO_2 _of greater than 90%, as measured by pulse oximetry. The interface chosen was facemask (Spectrum Reusable Full Face Mask; Respironics, Inc.). Weaning from NPPV was performed on a daily basis by gradually reducing pressure levels until adequate V_T _and V_e _levels could be reached and proper alveolar ventilation could be established. In the control group, invasive ventilation followed the previously administrated ICU ventilation support routine using Servo 900c or Servo 300 (Siemens AG, Munich, Germany) ventilators. Daily SBT was carried out thereafter in order to evaluate the possibility of extubation.

Both groups were monitored using a Hewlett-Packard monitor, which measured HR, f, SBP and DBP, and SpO_2 _by pulse oximetry continuously. They were followed up during the first 6 hours and then evaluated every 6 to 8 hours. Arterial gases were measured 2 hours after the patient was placed on ventilation and once a day until discontinuation of ventilation support. Data were collected by a team trained by one of the authors.

During follow-up of patients receiving IMV and NPPV, other complications were also described: pneumonia, sepsis, heart failure, tracheotomy, reintubation, and skin necrosis. Pneumonia was defined by clinical findings, new pulmonary infiltrate for longer than 48 hours after the patient was placed on that ventilation mode, and one or more of the following findings: purulent tracheal secretions, fever, and leukocytosis [20-22]. The clinical pulmonary infection score (CPIS) was also assessed on days 0 and 3, and pneumonia was diagnosed when CPIS was 7 or greater, according to the protocol followed in our service [23-25]. Sepsis was defined as a systemic inflammatory response syndrome (SIRS) associated with infection. SIRS was defined as a systemic inflammatory response to several severe clinical insults, which included two or more findings such as temperature of greater than 38°C or less than 36°C, HR of greater than 90 bpm, f of greater than 20 incursions per minute (ipm) or arterial partial pressure of carbon dioxide (PaCO_2_) of less than 32 mm Hg, and leukocyte count of greater than 12,000 cells per cubic millimeter, fewer than 4,000 cells per cubic millimeter, or greater than 10% of band cells [26]. Heart failure was defined clinically and radiographically by dyspnea with rales, S3, cardiomegaly, bilateral pulmonary edema, and elevated central venous pressure [27]. Tracheotomy was performed between 14 and 21 days after the beginning of IMV, according to our service's routine.

### Statistical analysis

Microsoft Excel 2000 software (Microsoft Corporation, Redmond, WA, USA) was used to store data. Statistical analysis was carried out using the Statistical Package for Social Sciences 12.0.1 (SPSS Inc., Chicago, IL, USA). The distribution of continuous variable frequencies was analyzed using means and standard deviations, which were compared using the Student *t *test. Discrete variables were evaluated using a contingency table and compared using the chi-square test. Significance level was established at a *P *value of less than 0.05.

## Results

**Figure 1:**
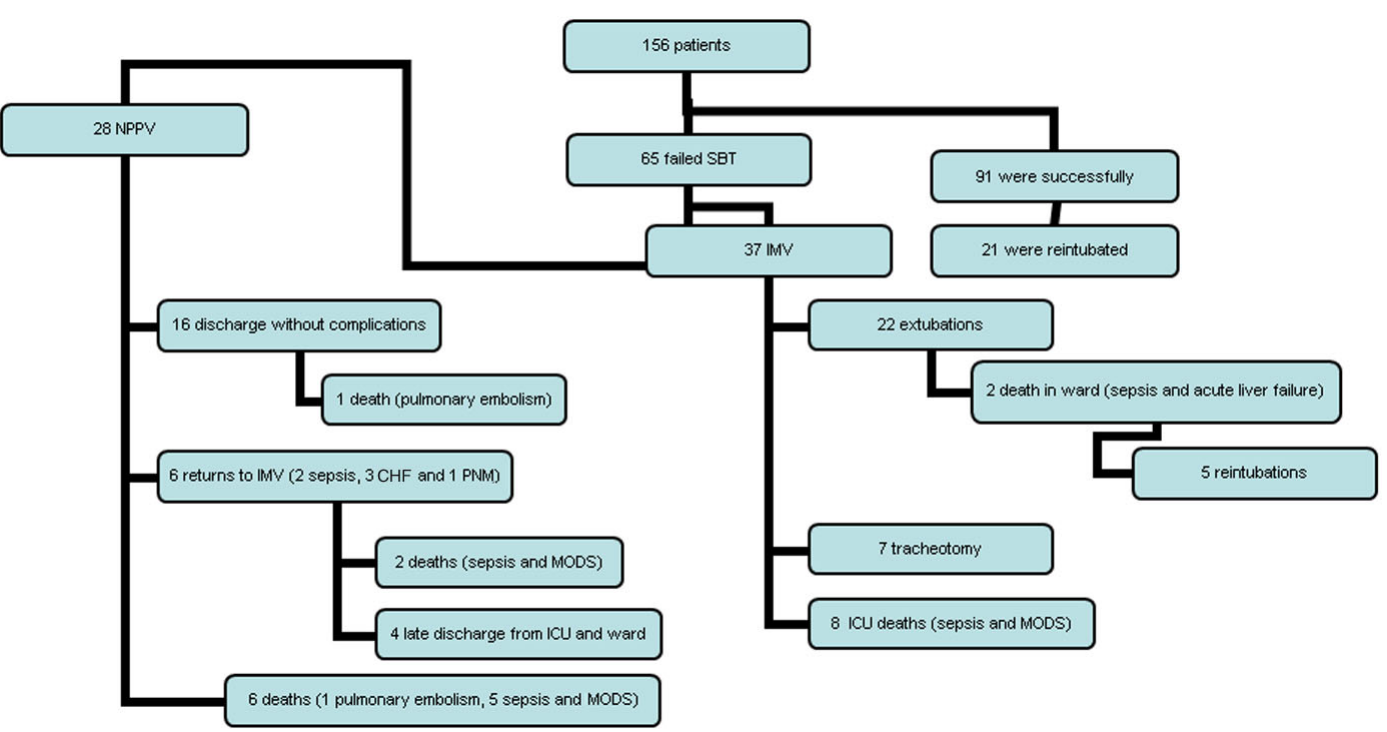
Flowchart showing the outcome of analyzed patients. DHOS, ICC, ; ICU, intensive care unit; IMV, invasive mechanical ventilation; MODS, multiple organ dysfunction syndrome; NPPV, noninvasive positive-pressure mechanical ventilation; PNM, pneumonia; SBT, spontaneous breathing T-piece trial.

Of the 156 patients submitted to SBT, 84 (53.8%) were randomly assigned to IMV and 72 (46.2%) to NPPV. After SBT, 91 patients were successfully extubated, but 26 (29.5%) had to be reintubated (Figure 1). Sixty-five patients (41.7%) failed SBT and were included in this study: 28 had been randomly assigned to NPPV and 37 to IMV. The patients in the NPPV group tended to be older. Other clinical characteristics were similar in the two groups. COPD aggravation, post-operative respiratory failure, and heart disease were the most frequent causes for the use of invasive ventilation support (Table 1) in both groups.

**Table 1:** Baseline characteristics of patients who failed spontaneous breathing trial

	NPPV (n = 28)	IMV (n = 37)	*P *value
Age, years	67.6 ± 15.5	59.7 ± 17.6	0.06
Gender, male/female	15/13	23/14	0.61
APACHE II score at admission	20 ± 6.8	18.0 ± 5.9	0.27
Duration of mechanical ventilation, days	7.3 ± 4.1	7.3 ± 4.1	0.98
Causes of mechanical ventilation, number (percentage)			
COPD aggravation and asthma	10 (35.6%)	13 (35.1%)	
Heart diseases	7 (25%)	4 (11%)	
Respiratory diseases	1 (3.6%)	2 (5.4%)	
Post-surgery respiratory failure	5 (18%)	11 (29.8%)	
Acute pulmonary lesion	0 (0%)	2 (5.4%)	
Pneumonia	3 (11%)	1 (2.7%)	
Tuberculosis	1 (3.6%)	2 (5.4%)	
Thoracic trauma	1 (3.6%)	1 (2.7%)	

Values are mean ± standard deviation or number (percentage). *P *value indicates comparison between treatment groups using *t *test or chi-square test. APACHE II, Acute Physiologic And Chronic Health Evaluation II; COPD, chronic obstructive pulmonary disease; IMV, invasive mechanical ventilation; NPPV, noninvasive mechanical ventilation.

The distribution of associated diseases was not significantly different between the NPPV and IMV groups, and the most frequent diseases were systemic hypertension (50% versus 27%), heart diseases (21.4% versus 21.6%), and diabetes mellitus (17.9% versus 21.6%). Moreover, respiratory characteristics of patients on mechanical ventilation, before the spontaneous breathing trial, were not statistically different between the groups, as shown in Table 2.

**Table 2:** Respiratory characteristics of patients before spontaneous breathing trial

	NPPV (n = 28)	IMV (n = 37)	*P *value
Respiratory rate, rpm	22.3 ± 4.2	21.2 ± 4.9	0.35
Tidal volume, mL	594 ± 0.21	629 ± 0.27	0.58
Peak inspiratory pressure, cm H_2_O	19.3 ± 4.9	18.6 ± 2.9	0.44
Maximal inspiratory pressure, cm H_2_O	36.0 ± 11.5	37.0 ± 16.1	0.64
Arterial pH	7.41 ± 0.07	7.41 ± 0.06	0.96
PaCO_2_, mm Hg	45.1 ± 11.5	40.1 ± 11.1	0.08
PaO_2_, mm Hg	88.7 ± 23.2	99.7 ± 29.5	0.11
SaO_2_, percentage	95.8 ± 3.1	96.6 ± 2.5	0.26

Values are mean ± standard deviation. *P *value indicates comparison between groups using *t *test. IMV, invasive mechanical ventilation; NPPV, noninvasive mechanical ventilation; PaCO_2_, arterial partial pressure of carbon dioxide; PaO_2_, arterial partial pressure of oxygen; rpm, respirations per minute; SaO_2_, arterial saturation of oxygen.

**Figure 2:**
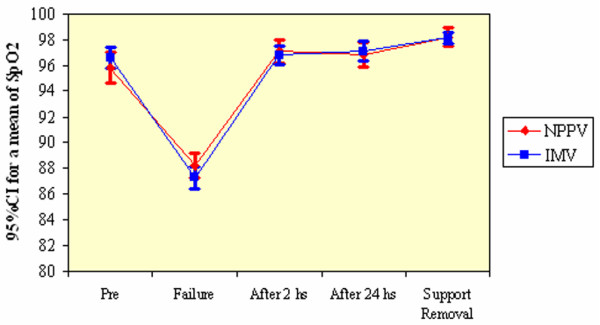
Changes in peripheral oxygen saturation (SpO_2_) in the two groups of patients. CI, confidence interval; IMV, invasive mechanical ventilation; NPPV, noninvasive positive-pressure mechanical ventilation.

During the spontaneous ventilation trial, 22 patients of the NPPV group and 20 of the IMV group were able to complete the test within 30 minutes and failed at 30 minutes, whereas 8 patients of the NPPV group and 17 of the IMV group failed before 30 minutes. The patient's final measurements were carried out at the failure moment. No statistically significant differences in cardiorespiratory parameters were found between groups at 1 minute or at the end of the trial, as shown in Table 3. Values of SpO_2 _measured during ventilation support were not statistically different between the two groups (Figure 2), which shows that both techniques were effective in keeping oxygenation.

**Table 3:** Cardiorespiratory parameters of patients during spontaneous breathing trial

	NPPV (n = 28)	IMV (n = 37)	PA	NPPV (n = 28)	IMV (n = 37)	PB
	First minute			Final		

Respiratory rate (f), ipm^a^	27.7 ± 5.7	30.05 ± 8.6	0.23	39.0 ± 2.8^b^	38.0 ± 3.1^b^	0.19
Tidal volume (V_T_), mL	389 ± 0.25	399 ± 0.28	0.51	278 ± 0.24^b^	268 ± 0.27^b^	0.46
f/V_T_	82.6 ± 45	87.6 ± 54.4	0.50	149.0 ± 67.9^b^	151.3 ± 58.4^b^	0.23
Heart rate, bpm^a^	95.7 ± 13.6	101.4 ± 20.3	0.57	108.4 ± 12.6^b^	116.1 ± 14.4^b^	0.77
SpO_2_^a^	95.1 ± 1.92	96.6 ± 1.97	0.15	88.2 ± 2.4	87.3 ± 2.6	0.09

Values are mean ± standard deviation. ^a^Values at moment of failure or at 30 minutes; ^b^*P *<0.05 (comparison between groups in the first and last minutes). bpm, beats per minute; ipm, incursions per minute; IMV, invasive mechanical ventilation; NPPV, noninvasive mechanical ventilation; PA, comparison between groups in the first minute (*t *test); PB, comparison between groups in the 30th minute (*t *test); SpO_2_, peripheral oxygen saturation.

The comparisons of gas measurements between the NPPV and IMV groups showed no significant differences. The pH values were as follows: before spontaneous breathing trial, 7.41 ± 0.07 for both groups; after up to 2 hours of spontaneous breathing trial, 7.39 ± 0.06 versus 7.40 ± 0.05; after 24 hours of ventilation support, 7.38 ± 0.08 versus 7.39 ± 0.07; and at the end of ventilation support removal, 7.38 ± 0.06 for both groups. PaCO_2 _before spontaneous ventilation trial for the two groups was 45.1 ± 11.5 versus 40.1 ± 11.1; up to 2 hours after failure, it was 43.2 ± 10.8 versus 41.6 ± 10.2; after 24 hours of support, it was 42.1 ± 11.3 versus 42.4 ± 11.2; and at final removal of ventilation support, it was 41.2 ± 10.9 versus 42.2 ± 10.8. PaO_2 _before spontaneous ventilation trial was 88.7 ± 23.2 versus 99.7 ± 29.5; after failure, it was 87.5 ± 22.4 versus 89.8 ± 25.1; after 24 hours of ventilation support, it was 88.6 ± 24.1 versus 92.5 ± 25.6; and at the removal of ventilation support, it was 89.2 ± 24.2 versus 95.5 ± 26.2.

Table 4 compares lengths of stay in the ICU and the hospital and mortality rate in both groups. Patients of the NPPV group had a shorter stay in the ICU and in the hospital. Duration of mechanical ventilation after random assignment amounted to 10.02 days for the IMV group and 7.5 days for the NPPV group. However, these differences were not statistically significant, even though the duration of mechanical ventilation was slightly reduced in the NPPV group. For the 6 patients returned to IMV, duration of mechanical ventilation amounted to 8 days. Mortality was similar in the two groups. Of the 28 patients in the NPPV group (Figure 1), 16 had no serious complications and were not on ventilatory support when discharged from the ICU. One of these patients died from pulmonary embolism. Six patients returned to invasive ventilation support because of abdominal sepsis (n = 2), worsening of congestive heart failure (n = 3), or pneumonia (n = 1). Two of these patients died, both due to sepsis and multiple organ dysfunction syndrome (MODS), whereas the remaining patients were discharged. Of the 37 patients in the IMV group (Figure 1), 8 patients died in the ICU due to sepsis and MODS and 2 died from kidney failure and sepsis. Discharged patients were not on ventilatory support when discharged from the ICU. However, 7 patients had to undergo tracheotomy and showed a greater incidence of complications, particularly infections. This higher rate of complications, chiefly pneumonia, in the IMV group, is shown in Table 5.

**Table 4:** Comparison of length of stay, death, causes of death, and mechanical ventilation time between groups

	NPPV (n = 28)	IMV (n = 37)	*P *value
Length of stay in ICU, days	18.9 ± 11.3	20.8 ± 10.9	0.51
Length of stay in hospital, days	9.6 ± 12.7	15.0 ± 18.6	0.19
Total length of stay in hospital, days	34.5 ± 20.6	42.4 ± 24.5	0.17
Death in ICU, number (percentage)	8 (28.6%)	8 (21.6%)	0.57
Death in ward, number (percentage)	1 (3.6%)	2 (5.4%)	1.00
Mechanical ventilation time after randomization, days	7.5 ± 7.8	10.0 ± 9.1	0.25
Total mechanical ventilation time, days	14.9 ± 9.9	17.3 ± 10.5	0.35

Values are mean ± standard deviation. *P *value indicates comparison between groups using chi-square test. ICU, intensive care unit; IMV, invasive mechanical ventilation; NPPV, noninvasive mechanical ventilation.

**Table 5:** Complications observed during the study

	NPPV (n = 28)	IMV (n = 37)	*P *value
Complications, number (percentage)	8 (28.6%)	28 (75.7%)	< 0.001
Type of complication, number (percentage)			
Pneumonia	1 (3.6%)	17 (45.9%)	< 0.001
Sepsis	2 (7.1%)	7 (18.9%)	0.28
Congestive heart failure	6 (21.4 %)	12 (32.4%)	0.41
Tracheotomy	0 (0%)	7 (18.9%)	0.01
Return to IMV	6 (21.4%)	-	
Skin necrosis	1 (3.6%)	-	

Values are mean ± standard deviation or number (percentage). *P *value indicates comparison between groups using chi-square test. IMV, invasive mechanical ventilation; NPPV, noninvasive mechanical ventilation.

## Discussion

The most important results of this study showed that, in patients who failed spontaneous ventilation trial when weaning was attempted, the combination of earlier tracheal extubation and NPPV ventilation support is a useful alternative. They decreased the incidence of pneumonia associated with mechanical ventilation, as well as the need for tracheotomy, in comparison with patients who were conventionally weaned from IMV.

Strong evidence supports the use of NPPV to avoid placement of an invasive airway and to reduce complications and mortality due to IMV [2,28,29]. However, few randomized clinical trials evaluated early use of NPPV to accelerate mechanical ventilation weaning. Older studies used this resource, but it was applied at a later stage in patients ventilated for a long time [30,31]. NPPV was recently used at earlier stages for mechanical ventilation weaning, and the results were favorable, particularly when used in selected patients, such as those with COPD and hypercapnic respiratory failure or respiratory acidosis [28,29]. Our study with a heterogeneous population of patients confirmed the beneficial effects of NPPV in comparison with IMV during weaning.

The process of gradual removal of mechanical ventilation poses an important clinical challenge, particularly in patients with pulmonary diseases, and its failure rates range from 35% to 67% [32]. Weaning failure during SBT in our study was not an infrequent clinical situation in patients on mechanical ventilation for an average of 7.3 days and was observed in 41.7% of the cases, which is in agreement with findings in the literature. Also in accordance with the literature, chronic pulmonary diseases were the most frequent causes of mechanical ventilation in our patients.

The NPPV group had shorter lengths of stay in the ICU and in the hospital, although this difference was not statistically significant. We did not observe any reductions in mortality, as did Ferrer and colleagues [14]. During the clinical course, the two treatment groups showed similar gas parameters, which is consistent with the literature [11]. In addition, there were no statistically significant differences in cardiorespiratory parameters measured in the first minute and at failure of the spontaneous breathing trial, which indicates that the groups had very similar baseline conditions. These findings indicate that NPPV is at least as safe a strategy as IMV.

There were significantly fewer complications in the NPPV group, with an important decrease in the incidence of pneumonia associated with mechanical ventilation and less need for tracheotomy. These results are similar to those of Nava and colleagues [11] and Ferrer and colleagues [14]. As in our study, those authors used NPPV after tracheal extubation and maintained it as long as necessary. On the other hand, Girault and colleagues [12] used intermittent periods of NPPV and did not observe any significant differences in the incidence of complications. The increased incidence of pneumonia which is observed in cases submitted to IMV for more than 3 days is associated with a high mortality rate [33-35]. Therefore, the decrease in its incidence, as observed in our study, is an important result. In addition, the decrease in the need for tracheotomy may result in fewer complications. These beneficial effects of NPPV, reducing the incidence of pneumonia and the need for tracheotomy, may also be correlated to cost reductions, but we did not analyze this possible correlation. It is important to note that the return to IMV of patients who did not benefit from the use of NPPV was due to aggravation of heart failure and abdominal sepsis and was not directly linked to the ventilation strategy used.

One of the limitations of this study was that our sample size was relatively small, though larger than samples in previous studies. A study with a greater number of patients might have yielded other significant results such as a reduction in the lengths of stay in the ICU and in the hospital. Also, although it is important to understand the role of NPPV in different groups of patients, a stratified analysis per subgroup was not possible because of the heterogeneity of our population as well as the small number of patients. Another limitation was that no data were collected after patients were discharged, and the analysis of late mortality was not possible. New studies should be carried out, with longer follow-up times and larger samples, to evaluate the effects of NPPV on the quality of life of patients on weaning ventilation support and to understand how the cause of ARF could affect the results of different weaning ventilation methods. Cost evaluation should also be included in these studies.

## Conclusion

The results of this study suggest that the combination of early extubation and NPPV is a good alternative for ventilation in a group of heterogeneous patients who initially failed weaning. NPPV use resulted in efficient gas exchange, a tendency to decrease ICU and hospital stays, and principally an important reduction in the incidence of pneumonia as well as in the need for tracheotomy when compared with conventional IMV weaning. Therefore, NPPV is a useful and safe strategy that may be considered during mechanical ventilation weaning.

## Key messages

• The combination of early extubation and noninvasive positive-pressure mechanical ventilation (NPPV) is a useful and safe alternative for ventilation in patients who fail initial weaning attempts.

• NPPV use resulted in efficient gas exchange, a tendency to decrease intensive care unit and hospital stays, and principally an important reduction in the incidence of pneumonia as well as in the need for tracheotomy.

## Abbreviations

ARF = acute respiratory failure; bpm = beats per minute; COPD = chronic obstructive pulmonary disease; CPIS = clinical pulmonary infection score; DBP = diastolic blood pressure; FiO_2 _= fraction of inspired oxygen; HR = heart rate; ICU = intensive care unit; IMV = invasive mechanical ventilation; MODS = multiple organ dysfunction syndrome; NPPV = noninvasive positive-pressure mechanical ventilation; PaCO_2 _= arterial partial pressure of carbon dioxide; PaO_2 _= arterial partial pressure of oxygen; PEEP = positive end-expiratory pressure; PI_max _= maximal inspiratory pressure; PTPdi = diaphragmatic pressure-time product; f = respiratory rate; f/V_T _= respiratory rate to tidal volume ratio; SBP = systolic blood pressure; SBT = spontaneous breathing T-piece trial; SIRS = systemic inflammatory response syndrome; SpO_2 _= peripheral oxygen saturation; V_e _= minute volume; V_T _= tidal volume.

## Competing interests

The authors declare that they have no competing interests.

## Authors' contributions

CT and SV made substantial contributions to the study conception and design, analysis and interpretation of data, as well as drafting of the manuscript. The Research Group in Mechanical Ventilation Weaning was responsible for data collection. Both authors read and approved the final manuscript.
